# Association between right ventricular dysfunction and adverse cardiac events in mild COPD patients

**DOI:** 10.1111/eci.13887

**Published:** 2022-10-17

**Authors:** Giuseppe Armentaro, Corrado Pelaia, Velia Cassano, Sofia Miceli, Raffaele Maio, Maria Perticone, Daniele Pastori, Pasquale Pignatelli, Francesco Andreozzi, Francesco Violi, Giorgio Sesti, Angela Sciacqua

**Affiliations:** ^1^ Department of Medical and Surgical Sciences University Magna Græcia Catanzaro Italy; ^2^ Department of Health Sciences University Magna Græcia Catanzaro Italy; ^3^ Department of Clinical Internal, Anesthesiological and Cardiovascular Sciences Sapienza University of Rome Rome Italy; ^4^ Department of Clinical and Molecular Medicine Sapienza University of Rome Rome Italy

**Keywords:** COPD, MACE, oxidative stress, right heart, TAPSE

## Abstract

**Background:**

Lung hyperinflation and systemic inflammation are currently believed to be the most important causes of right heart alterations in chronic obstructive pulmonary disease (COPD) patients. A multicentre observational study was performed to assess the morphological and functional parameters of right ventricle (RV) in COPD subjects, as well as to evaluate the potential prognostic impact on the development of major cardiovascular adverse events (MACEs).

**Methods:**

For this retrospective study, from 1 January 2010 to 31 December 2021, we enrolled COPD patients on the basis of their airflow limitation. In particular, we selected subjects spanning across GOLD 1 and 2 functional stages. Clinical, laboratory and functional parameters were collected at baseline. Echocardiography was routinely performed in all COPD patients. RV dysfunction was defined on the basis of tricuspid annular plane systolic excursion (TAPSE) values. MACE occurrence (non‐fatal ischemic stroke, non‐fatal myocardial infarction, cardiac revascularization or coronary bypass surgery and cardiovascular death) was evaluated during a median follow‐up of 55 (36–72) months.

**Results:**

Among the 749 enrolled patients, 408 subjects had a TAPSE value ≥20 mm, while the remaining 341 had a TAPSE value <20 mm. In patients with TAPSE ≥20 mm the observed MACEs were 1.9 events/100 patient‐year, while in the group with a worse right heart function there were 4.2 events/100 patient‐year (*p* < .0001). The multivariate analysis model confirmed the association between RV dysfunction and MACE. Indeed, a 1‐mm increase in TAPSE value and the intake of long‐acting β_2_‐receptor agonists (LABA)/long‐acting muscarinic antagonist (LAMA) inhaled therapy were protective factors for the onset of MACE, while the presence of diabetes mellitus and high values of both uric acid (UA) and systolic pulmonary arterial pressure (S‐PAP) enhanced the risk of MACE in study participants.

**Conclusions:**

The results of this study showed that in patients with mild COPD there is an association between right heart dysfunction and the risk of MACE during follow‐up.

## Graphical abstract

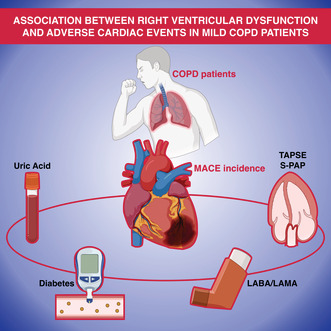

## 1 INTRODUCTION

Chronic obstructive pulmonary disease (COPD) is a preventable and treatable respiratory syndrome, which represents one of the major causes of morbidity and mortality worldwide.^1^, ^2^ During the last decade, it has been estimated that the potential loss of life years due to COPD increased by 13.2%, with relevant social and economic consequences.^3^ Globally, the cost of COPD is expected to enhance in the near future due to both ageing and risk factors.^4^ COPD management is often complicated by the presence of several comorbidities; in particular, cardiovascular disorders represent the most frequent systemic manifestations which significantly have an impact on COPD progression, clinical outcomes, mortality and economic burden.^5^, ^6^ The hypothesis that COPD itself may represent a cardiovascular risk factor has also gained a remarkable consideration.^7^ The pathogenic mechanisms linking COPD and heart diseases are not well understood, even if lung hyperinflation and systemic inflammation are currently believed to be the most important causes.^8^ Indeed, because of the anatomical and functional interactions between pulmonary and cardiovascular systems, many dysfunctions affecting each of these two districts often extend to the other one.^9^ In particular, right heart alterations frequently occur in COPD patients.^10^ When pulmonary vascular pressure enhances gradually and progressively, the main impairment of RV is hypertrophy, which occurs during the initial stages of chronic cor pulmonale (CCP).^11^

Although the underlying causes may be different, the common pathophysiologic factor leading to CCP is pulmonary hypertension (PH). PH is often induced by COPD, idiopathic pulmonary fibrosis or chronic pulmonary thromboembolism. Among the different mechanisms underpinning an increase in pulmonary vascular resistance (PVR), the most studied is alveolar hypoxia. In this regard, pulmonary vasoconstriction is the result of either acute hypoxia or vascular structural remodelling due to hypoxaemia.^12^ Hypoxaemia can also contribute to the increase in PVR indirectly, through the induction of polycythaemia and the release of inflammatory mediators. Mechanical factors such as vascular bed loss in patients with emphysema, thrombosis and hyperinflation also contribute to the increase in pulmonary artery pressure.^13^, ^14^ While the diagnosis of cor pulmonale should be based on catheterization of the right heart, routine evaluation of pulmonary artery pressure is commonly performed by means of colour Doppler echocardiogram, which represents a non‐invasive and reliable diagnostic tool.^15^

Severity of pulmonary hypertension and development of cor pulmonale are two of the main factors involved in the mortality of patients with COPD. It has been observed that in these patients the onset of chronic cor pulmonale reduces survival up to 30%^16^ and is also associated with a higher incidence of exacerbations.^17^ The results of a recent study suggest greater impairment of right ventricular function in patients with more severe airflow limitation.^18^ Indeed, in subjects with severe COPD right ventricular dysfunction has been shown to be one of the independent predictors of survival, together with impaired arterial oxygen partial pressure and pulmonary function.^19^ However, to our knowledge, no data are currently available with regard to the association between right ventricular dysfunction and adverse cardiac events in patients with COPD at early disease stages.

The main purpose of our present study was to assess the morphological and functional parameters of the right ventricle (RV) in COPD patients at early disease stages (Global initiative for chronic obstructive lung disease, GOLD, stages 1 and 2), as well as to evaluate the potential prognostic impact on the development of major cardiovascular adverse events (MACEs) during follow‐up.

## 2 METHODS

### 2.1 Study population

In the present retrospective study, subjects of Caucasian ethnicity with COPD (GOLD stages 1 and 2) were recruited from 1 January 2010 to 31 December 2021 at the Clinic Units of Geriatrics and Respiratory Diseases of ‘Magna Graecia’ University Hospital of Catanzaro, Italy, and at the First Internal Medicine Unit of Sapienza University of Rome, Italy. A careful medical history was collected in all subjects by evaluating comorbidities, cardiovascular risk factors and drug therapy. A complete cardiovascular physical examination was carried out, and both body weight and body mass index (BMI) were also measured. Blood samples were collected for the determination of several laboratory parameters (see next paragraph). Spirometry and cardiac ultrasonography were also carried out. In particular, in our clinical practice echocardiography is routinely performed in all COPD patients referring to the above centres, with the aim of assessing the eventual impact of this respiratory disease on cardiac function.

We selected study participants under bronchodilator treatment, according to their stratification across GOLD 1 and 2 functional stages. Patients included within GOLD stages 3 and 4 were not considered in this investigation, because our aim was to detect early COPD subjects with RV dysfunction before the occurrence of a severe worsening of their airflow limitation. Individuals with active cancer, heart failure with reduced or mid‐range ejection fraction (left ventricular ejection fraction <50%), advanced liver cirrhosis (Child‐Pugh C) and renal insufficiency (baseline estimated glomerular filtrate‐eGFR <30 ml/min/1.73 m^2^) were excluded from the study. In addition, other exclusion criteria were the presence of inflammatory bowel disease or systemic autoimmune disorders (e.g., rheumatoid arthritis, systemic lupus erythematosus, scleroderma and dermatomyositis). Reporting of the study conforms to broad EQUATOR guidelines.^20^

### 2.2 Laboratory parameters

All laboratory measurements were performed after at least 12 h of fasting. Glycaemia was determined by the glucose oxidase method (glucose analyser, BeckmanCoulter, Milan). Blood levels of total cholesterol, low‐density lipoprotein (LDL) cholesterol, high‐density lipoprotein (HDL) cholesterol and triglycerides were analysed by enzymatic methods (Roche Diagnostics GmbH, Mannheim, Germany). Creatinine levels were measured using the Jaffe method. The estimation of glomerular filtration rate (eGFR) was based on the new CKD‐EPI (Chronic Kidney Disease Epidemiology Collaboration) equation.^21^ Serum uric acid (UA) levels were assessed using URICASE/POD method (Boehringer Mannheim, Mannheim, Germany). The high‐sensitivity C‐reactive protein (hs‐CRP) was quantified by the immunoturbidimetric method automated system (Cardio Phase hs‐CRP, Milan, Italy).

### 2.3 Echocardiographic parameters

Standard left ventricular ultrasonography in both M‐mode (motion mode) and B‐mode (two‐dimensional mode) was performed in all patients, according to the recommendations of the American Society of Echocardiography (ASE).^22^ Recordings were made using a VIVID 7 Pro ultrasound system (GE Technologies, Milwaukee, Wisconsin, USA) and a 2.5 MHz transducer. Echocardiographic parameters at baseline and during the follow‐up were detected by expert operators, in order to minimize measurement errors. However, the operator was not aware of patient's clinical data, and the values represented the average of at least three measurements. Among the parameters of left ventricular global systolic function, left ventricular ejection fraction (LVEF), cardiac output (CO) and cardiac index (CI) were evaluated. LVEF was calculated by the Simpson biplane method. Both volumes were then indexed for body surface area and expressed as ml/m^2^. Left atrial diameter (LAD) was assessed using M‐mode or two‐dimensional echocardiography. Right ventricular systolic parameters were also measured, by estimating the systolic pulmonary arterial pressure (S‐PAP).^22^ Tricuspid regurgitant velocity (TRV) was analysed by continuous Doppler at level of the atrioventricular plane of the tricuspid valve, in projection with the four apical chambers or, in the case of eccentric jets, in parasternal short axis: therefore, S‐PAP was derived through the Bernoulli equation. Diastolic dysfunction was detected by recording pulse‐wave Doppler patterns at the mitral valve, in order to measure early (E) and late (A) diastolic filling velocities from the 4‐chamber view. Left ventricular mass was calculated using the Devereux equation and was subsequently expressed as left ventricular mass index (LVMI). Tissue Doppler imaging (TDI) was performed at the junction of the septal and lateral mitral annulus. The diameter of the right ventricular outflow tract (RVOT) and the right atrium area (RAA) were obtained according to ASE recommendations.^22^ The movement of the tricuspid annulus was recorded at the free wall of the RV for the tricuspid annular plane systolic excursion (TAPSE), which expresses the right longitudinal function. PVR was estimated as previously described.^23^ In addition, for a more complete assessment of right ventricular function, the TAPSE/S‐PAP ratio, an index of the right ventricular length/strength relationship, and right ventricular ejection efficiency (RVEe) were also calculated.^24^, ^25^, ^26^ RVFAC was obtained from a four‐chamber view in which the right ventricle end‐diastolic area (RVEDA) and right ventricle end‐systolic area (RVESA) were measured.^24^

### 2.4 Cardiovascular endpoints

MACE and total mortality were identified as study endpoints. MACE included non‐fatal ischemic stroke, non‐fatal myocardial infarction (MI), coronary revascularization (i.e., percutaneous coronary intervention or coronary artery bypass grafting) and cardiovascular death. The diagnosis of myocardial infarction was made according to the universal definition proposed by the Joint ESC/ACCF/AHA/WHF.^27^ If a patient died within four weeks after a stroke or MI, this event was classified as fatal. Cardiovascular death included sudden death, progressive heart failure and death related to surgical or percutaneous revascularization procedures. The diagnosis of ischemic stroke was determined by clinical manifestations and confirmed by radiological findings.^28^ Data on MACE were collected during the follow‐up. When an event occurred, a standardized form was filled out by the examiners. Details of each event were recorded, as well as death certificates, hospital discharge letter or copy of hospitalization medical record, and other clinical documentation obtained from patients or their relatives. Evaluation of cardiovascular events was performed by a committee composed of physicians who did not participate in patient recruitment (R.M., M.P., D.P., F.A., and A.S.). Each member of the committee assessed and judged the events independently and blindly.

### 2.5 Statistical analysis

Data were expressed as mean ± standard deviation (SD) for normally distributed data, as median and interquartile range (IQR) for data not normally distributed, and as number and percentage for categorical variables. Student's *t*‐test was performed for unpaired data for continuous variables, Wilcoxon's test for unpaired data for non‐continuous variables and *χ* ^2^ tests for categorical variables. The overall population was divided into two groups based on the TAPSE value (20 mm) as the main parameter of right ventricular function. The accuracy of the TAPSE parameter as a predictor of the onset of MACE, both as a continuous and categorical variable, was evaluated by processing a receiver operating characteristic (ROC) curve. The area under the curve (AUC) described how measure the value of TAPSE was associated with the onset of events. The incidence of events was calculated as the number of events per 100 patient‐year. Since the follow‐up was not uniform for all patients, the onset of MACE was not assessed at the same time, but a regression analysis based on the Cox proportional model was used, correcting the analysis for possible covariates associated with the finding of MACE. In particular, a univariate Cox regression model was performed on the incidence of MACE; subsequently, the variables that significantly correlated with the appearance of MACE were included in a multivariate Cox regression model to calculate the hazard ratio (HR) for the independent predictors associated with the incidence of MACE. The differences were considered statistically significant for *p* value <.05. All analyses were performed using the SPSS 26.0 statistical program for Windows (SPSS Inc., Chicago, IL, USA).

## 3 RESULTS

**FIGURE 1:**
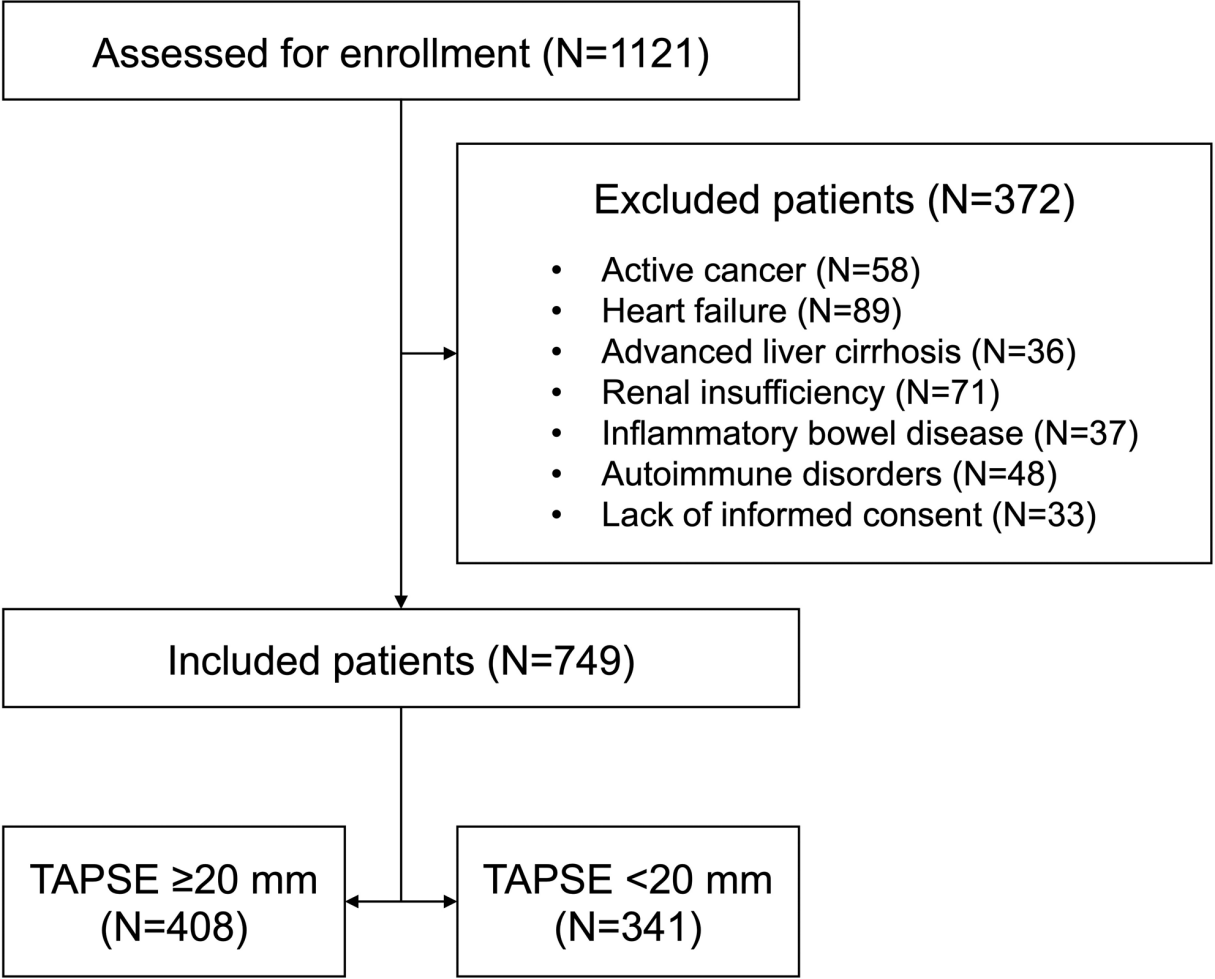
Flow diagram showing the process of patient inclusion. TAPSE, tricuspid annular plane systolic excursion.

Overall, 1121 COPD patients (GOLD stages 1 and 2) were screened. Among these, 749 subjects met the inclusion criteria, whereas 372 were excluded because of several reasons (Figure 1). Among the 749 enrolled patients, of which 351 males and 398 females, with an average age of 62.3 ± 10.2 years, 408 had a TAPSE value ≥20 mm (above the median; first group), while the remaining 341 had a TAPSE value <20 mm (below the median; second group). The median duration of follow‐up was 55 (36–72) months.

Table 1 shows the anthropometric, hemodynamic, biohumoral and clinical characteristics of the entire study population, in accordance with the median value of TAPSE.

**TABLE 1:** Anthropometric, hemodynamic and biohumoral characteristics, stratified according to median value of TAPSE

	All	TAPSE ≥20 mm	TAPSE <20 mm	*p*
	(*n* = 749)	(*n* = 408)	(*n* = 341)	
Gender, m/f	351/398	180/228	171/170	.099
Age, years	62.3 (10.2)	62.9 (10.1)	61.7 (10.3)	.098
BMI, kg/m^2^	29.2 (5.7)	29.3 (5.6)	29.1 (5.8)	.440
SBP, mmHg	140.1 (15.7)	139.9 (15.5)	140.4 (16.0)	.850
DBP, mmHg	82.1 (10.7)	81.6 (10.5)	82.6 (10.9)	.312
Pulse pressure, mmHg	58.0 (14.2)	58.3 (14.0)	57.8 (14.5)	.662
Fasting glucose, mg/dl	104.3 (23.2)	105.7 (22.8)	102.8 (23.7)	.798
LDL‐cholesterol, mg/dl	115.5 (35.5)	115.3 (35.2)	115.9 (36.0)	.990
Triglycerides, mg/dl	120.0 (86–164)	120 (85–169.5)	117 (87–159)	.639
eGFR, ml/min/1.73 m^2^	79.7 (26.0)	80.1 (25.1)	79.2 (27.0)	.244
Uric acid, mg/dl	5.5 (2.4)	5.6 (2.4)	5.4 (2.3)	.411
hs‐CRP, mg/dl	2.5 (1.7–3.4)	2.5 (1.6–3.4)	2.5 (1.7–3.4)	.201
FEV_1_, %	82.4 (21.8)	85.7 (21.6)	78.4 (21.6)	.449
COPD exacerbations, *n* (%)	77 (10.2)	38 (9.3)	39 (11.4)	.340
Follow‐up duration, months	55 (36–72)	56 (36–74.5)	54 (35–70)	.684

Abbreviations: BMI, body mass index; COPD, chronic obstructive pulmonary disease; DBP, diastolic blood pressure; eGFR, estimated glomerular filtration rate; FEV_1_, forced expiratory volume in the 1st second; hs‐CRP, high‐sensitivity C‐reactive protein; LDL, low‐density lipoprotein; SBP, systolic blood pressure; TAPSE, tricuspid annular plane systolic excursion.

Table 2 shows the prevalence of the different cardiovascular risk factors and therapies used in the entire study population affected by COPD, in accordance with the median value of TAPSE. Statistically significant differences between the two groups were observed for smoking habit (67.4% in the TAPSE <20 mm group and 56.6% in the TAPSE ≥20 mm group, *p* = .034), and inhalation therapy with either long‐acting muscarinic antagonist (LAMA) (35.2% in the TAPSE <20 mm group and 44.1% in the TAPSE ≥20 mm group, *p* = .013) or long‐acting β_2_‐receptor agonists (LABA)/LAMA associations (38.4% in the TAPSE <20 mm group and 23.0% in the TAPSE ≥20 mm group, *p* = .0001). On the other hand, they did not differ with regard to the assumption of monotherapy with LABA (*p* = .054).

**TABLE 2:** Cardiovascular risk factors and therapies, stratified according to median value of TAPSE

	All	TAPSE ≥20 mm	TAPSE <20 mm	*p*
	(*n* = 749)	(*n* = 408)	(*n* = 341)	
Smokers, *n* (%)	461 (61.5)	231 (56.6)	230 (67.4)	.034
Hypertension, *n* (%)	577 (77.0)	304 (74.5)	273 (80.0)	.072
Obesity, *n* (%)	270 (36.0)	147 (36.0)	123 (36.1)	.990
Hypercholesterolemia, *n* (%)	199 (26.5)	117 (28.7)	82 (24.0)	.153
Ischemic heart disease, *n* (%)	274 (36.6)	147 (36.0)	127 (37.2)	.731
Atrial fibrillation, *n* (%)	182 (24.3)	93 (22.8)	89 (26.1)	.293
Diabetes, *n* (%)	202 (26.9)	103 (25.2)	99 (29.0)	.244
Chronic kidney disease, *n* (%)	157 (20.9)	77 (18.9)	80 (23.4)	.124
Drugs				
Antihypertensives, *n* (%)	577 (77.0)	304 (74.5)	273 (80.0)	.072
RAAS inhibitors, *n* (%)	509 (67.9)	82 (69.1)	227 (66.5)	.456
Calcium‐blockers, *n* (%)	261 (34.8)	143 (35.0)	118 (34.6)	.898
Diuretics, *n* (%)	308 (41.1)	171 (41.9)	137 (40.2)	.630
Beta blockers, *n* (%)	70 (9.3)	39 (9.5)	31 (9.1)	.826
Antiplatelet agents, *n* (%)	184 (24.5)	93 (22.8)	91 (26.7)	.217
Anticoagulants, *n* (%)	38 (5.1)	20 (4.9)	18 (5.2)	.815
Statins, *n* (%)	199 (26.5)	117 (28.7)	82 (24.0)	.153
LAMA, *n* (%)	300 (40.0)	180 (44.1)	120 (35.2)	.013
LABA, *n* (%)	224 (29.9)	134 (32.8)	90 (26.4)	.054
LABA/LAMA, *n* (%)	225 (30.0)	94 (23.0)	131 (38.4)	<.0001

Abbreviations: LABA, long‐acting β_2_‐receptor agonists; LAMA, long‐acting muscarinic antagonist; RAAS, renin angiotensin aldosterone system; TAPSE, tricuspid annular plane systolic excursion.

Table 3 shows the echocardiographic parameters stratified by the median value of TAPSE. In particular, between the first group and the second one statistically significant difference were found for TAPSE, right ventricular end‐diastolic diameter (RVEDD), RVFAC, right E/A and RVEe. No statistically significant differences were found in terms of left E/A, left E/E, S‐PAP, RAA, RVEDA, RVESA and PVR.

**TABLE 3:** Echocardiographic parameters, stratified according to median value of TAPSE

	All	TAPSE ≥20 mm	TAPSE <20 mm	*p*
	(*n* = 749)	(*n* = 408)	(*n* = 341)	
LAVI, ml/m^2^	25.8 (7.5)	26.4 (8.0)	27.2 (6.5)	<.0001
LVMI, g/m^2^	135.7 (38.1)	133.1 (36.7)	138.6 (39.5)	.027
Left E/A	0.94 (0.87)	0.97 (0.3)	0.91 (0.28)	.292
Left E/e'	9.9 (2.9)	9.4 (2.9)	10.5 (2.8)	.577
TAPSE, mm	20.5 (3.9)	23.1 (3.1)	17.3 (2.0)	<.0001
S‐PAP, mmHg	32.2 (8.6)	30.7 (8.2)	34.0 (8.8)	.247
TAPSE/S‐PAP, mm/mmHg	0.83 (0.46)	0.92 (0.36)	0.72 (0.53)	.002
S′, m/s	0.08 (0.02)	0.08 (0.02)	0.08 (0.02)	.558
RAA, cm^2^	18.5 (4.7)	18.1 (4.6)	19.1 (4.7)	.264
RVEDD, cm	2.3 (0.4)	2.3 (0.3)	2.4 (0.4)	.009
RVEDA, cm^2^	18.0 (4.6)	17.6 (4.8)	18.4 (4.3)	.405
RVESA, cm^2^	10.4 (3.0)	10.0 (2.9)	11.0 (3.0)	.085
RVFAC, %	41.9 (8.6)	43.0 (8.9)	40.5 (8.0)	.005
Right E/A	1.03 (0.33)	1.04 (0.32)	1.02 (0.35)	.015
Right E/e'	6.4 (2.0)	6.09 (1.99)	6.9 (2.1)	.055
PVR, Woods	1.9 (0.5)	1.8 (0.5)	2.0 (0.4)	.330
RVEe	11.5 (4.8)	13.7 (4.9)	8.8 (2.8)	<.0001

Abbreviations: LAVI, left atrium volume index; LVMI, left ventricular mass index; PVR, pulmonary vascular resistance; RAA, right atrium area; RVEDA, right ventricle end‐diastolic area; RVEDD, right ventricular end‐diastolic diameter; RVEe, right ventricular ejection efficiency; RVESA, right ventricle end‐systolic area; RVFAC, right ventricular fractional area change; S‐PAP, systolic pulmonary arterial pressure; TAPSE, tricuspid annular plane systolic excursion.

During a median follow‐up of 55 (36–72) months, a total of 77 exacerbations (2.13 events/100 patient‐year) were observed, including 38 in the group of patients with TAPSE ≥20 mm (1.88 events/100 patient‐year) and 39 in the group of patients with TAPSE <20 mm (2.45 events/100 patient‐year) (*p* = .340).

**FIGURE 2:**
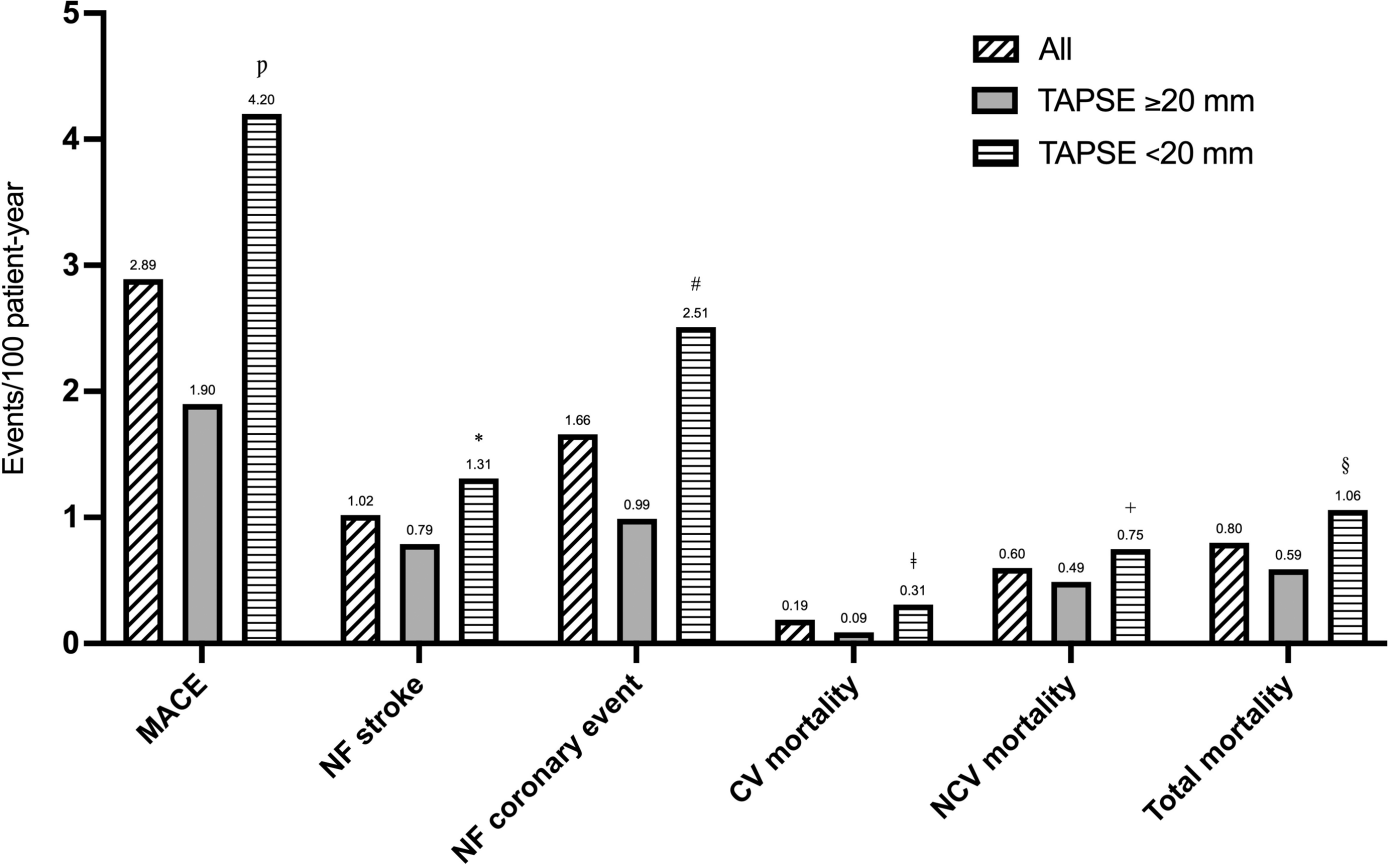
Incidence of different MACEs in COPD patients during follow‐up, stratified according to median value of TAPSE. CV, cardiovascular; MACE, major cardiovascular adverse events; NF, non‐fatal; NCV, non‐cardiovascular; TAPSE, tricuspid annular plane systolic excursion. ^Ƿ^ *p* = <.0001; *p = .154; ^#^ *p* = .0006; ^ǂ^ *p* = .166; ^+^ *p* = .388; ^§^ *p* = .148.

A total of 104 MACE were observed (2.89 events/100 patient‐year). Of these, 60 (57.7%) were non‐fatal coronary events (1.66 events/100 patient‐year), and 37 (35.6%) were non‐fatal cerebrovascular events (1.02 events/100 patient‐year) (Figure 2). In particular, in patients with TAPSE ≥20 mm the observed MACE were 38 (1.9 events/100 patient‐year) while in the group with a worse right heart function there were 66 (4.2 events/100 patient‐year) (*p* < .0001); non‐fatal coronary events were 20 (52.6%) in the first group (0.99 events/100 patient‐year) and 40 (60.6%) in the second group (2.51 events/100 patient‐year) (*p* = .0006), while non‐fatal cerebrovascular events were 16 (42.1%) (0.79 events/100 patient‐year) in the first group and 21 (31.8%) (1.31 events/100 patient‐year) in the second group (*p* = .154) (Figure 2). Regarding the whole number of deaths, these were 12 (0.59 events/100 patient‐year) in patients with TAPSE ≥20 mm, and 17 (1.06 events/100 patient‐year) in patients with TAPSE <20 mm (*p* = .148). Cardiovascular deaths were 2 (16.7%) (0.09 events/100 patient‐year) in the first group and 5 (29.4%) (0.31 events/100 patient‐year) in the second one (*p* = .166) (Figure 2).

**FIGURE 3:**
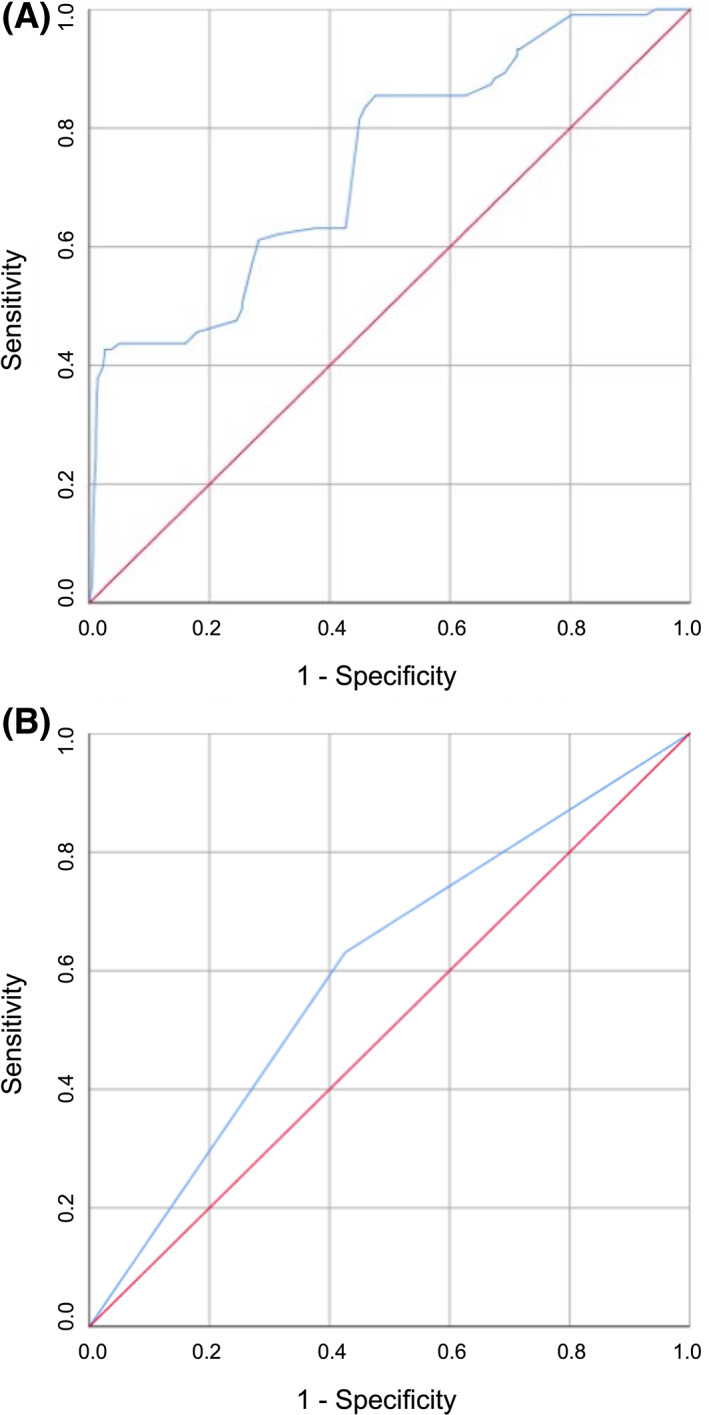
Analysis of ROC curves for the identification of MACE, stratified according to TAPSE value expressed as continuous variable (A) and as dichotomous variable (B). AUC, area under the curve; MACE, major cardiovascular adverse events; ROC, receiver operating characteristic; TAPSE, tricuspid annular plane systolic excursion.

**FIGURE 4:**
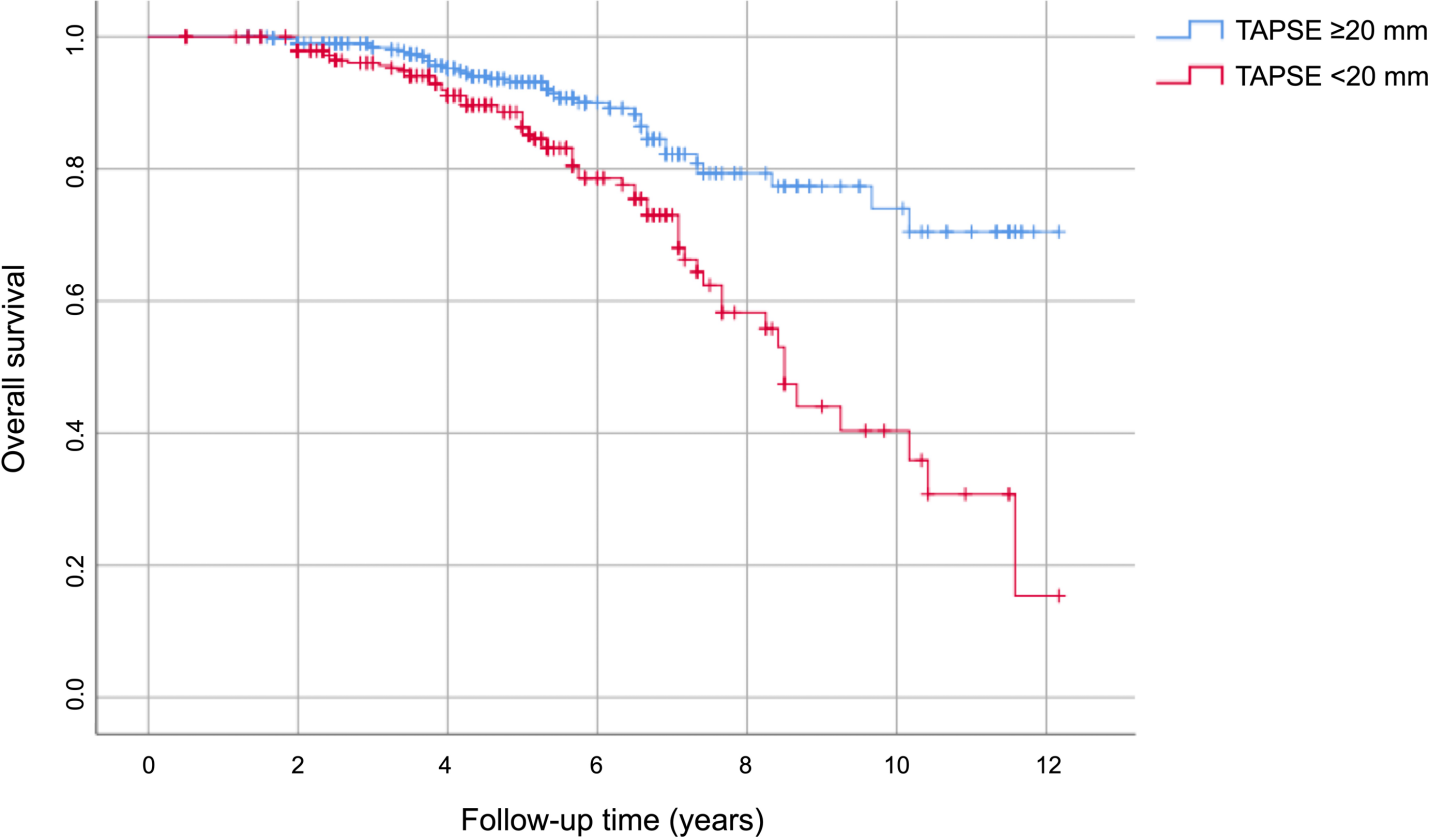
Kaplan–Meier survival curves, stratified according to median value of TAPSE. TAPSE, tricuspid annular plane systolic excursion.

AUC was used to evaluate the accuracy of TAPSE as a predictive value of the onset of MACE both as a continuous variable (A) and as a dichotomous variable above and below the median (B). Figure 3A shows the ROC curve of TAPSE as a continuous variable; TAPSE as a continuous variable has a greater discriminating power in predicting the development of MACE (AUC 0.741; standard error 0.027; 95% CI 0.688–0.794; *p* < .0001), compared with TAPSE as a dichotomous value (AUC 0.602; standard error 0.030; 95% CI 0.544–0.661; *p* = .001) (Figure 3B). Figure 4 shows the survival curves for the two different subgroups.

From the results of Cox linear regression analysis of the incidence of MACE, a statistically significant association was found with 1 mm increase in TAPSE value, 5 mmHg increase in S‐PAP and 1 mg/dl increase in UA values, presence of obesity, diabetes mellitus and exacerbations, intake of statins and dual inhalation therapy with LABA/LAMA (Table 4). The variables found to be significantly associated with the onset of MACE in the Cox univariate analysis were included in a multivariate analysis model to define the independent predictors of MACE (Table 5). In particular, a 1 mm increase in TAPSE value reduced the risk of MACE by 33%. Moreover, in comparison with single bronchodilator treatment, LABA/LAMA inhaled therapy was associated with a 19.7% risk reduction in MACE. The presence of diabetes mellitus increased the risk of MACE by 86%, while a 1 mg/dl‐increment in the UA value enhanced this risk by 12.5%. Finally, an increase in S‐PAP value of 5 mmHg correlated with a 22% risk enhancement of MACE.

**TABLE 4:** Univariate linear regression analysis focused on MACE incidence

	Hazard ratio	95% confidence interval	*p*
*Clinical parameters*			
Age, 10 years	0.848	0.612–1.174	.321
Gender, m/f	1.646	0.933–2.902	.085
Obesity, yes/no	1.796	1.017–3.172	.044
Hypertension, yes/no	1.434	1.007–4.562	.078
Ischemic heart disease, yes/no	1.379	0.550–3.457	.494
Diabetes, yes/no	2.288	1.278–4.095	.005
Statins, yes/no	0.459	0.221–0.953	.037
Atrial fibrillation, yes/no	1.693	0.953–3.006	.072
COPD exacerbations, yes/no	2.148	1.070–4.311	.032
LABA/LAMA, yes/no	0.791	0.685–0.974	<.0001
FEV_1_, %	1.014	0.995–1.034	.153
*Biological parameters*			
Baseline eGFR, 10 ml/min/1.73 m^2^	0.992	0.874–1.126	.897
LDL‐cholesterol, 10 mg/dl	0.946	0.825–1.085	.427
Uric acid, 1 mg/dl	1.142	1.006–1.296	.040
*Echocardiographic parameters*			
LAVI, ml/m^2^	0.971	0.929–1.016	.205
LVMI, g/m^2^	1.001	0.994–1.008	.791
Left E/e'	1.072	0.983–1.170	.116
TAPSE, 1 mm	0.625	0.556–0.939	<.0001
S‐PAP, 5 mmHg	1.521	1280–1808	<.0001
PVR, 1 Wood	1.421	0.785–2.573	.246

Abbreviations: COPD, chronic obstructive pulmonary disease; eGFR, estimated glomerular filtration rate; FEV_1_, forced expiratory volume in the 1st second; LABA, long‐acting β_2_‐receptor agonists; LAMA, long‐acting muscarinic antagonist; LAVI, left atrium volume index; LDL, low‐density lipoprotein; LVMI, left ventricular mass index; MACE, major cardiovascular adverse events; PVR, pulmonary vascular resistance; S‐PAP, systolic pulmonary arterial pressure; TAPSE, tricuspid annular plane systolic excursion.

**TABLE 5:** Multivariate regression analysis focused on MACE incidence

	Hazard ratio	95% confidence interval	*p*
TAPSE, 1 mm	0.665	0.562–0.942	<.0001
LABA/LAMA, yes/no	0.803	0.697–0.985	<.0001
Uric acid, 1 mg/dl	1.125	1.039–1.218	.041
S‐PAP, 5 mmHg	1.220	1.073–1.338	<.0001
Diabetes, yes/no	1.859	1.249–2.767	.025

Abbreviations: LABA, long‐acting β_2_‐receptor agonists; LAMA, long‐acting muscarinic antagonist; MACE, major cardiovascular adverse events; S‐PAP, systolic pulmonary arterial pressure; TAPSE, tricuspid annular plane systolic excursion.

## 4 DISCUSSION

The results of this study, conducted on a large population of patients with COPD (GOLD stages 1 and 2) and several cardiovascular risk factors, showed that there is an association between a good function of the right heart and a lower incidence of the main cardiovascular adverse events during follow‐up. In fact, in the entire population of COPD patients, it emerged that a higher TAPSE value, and therefore a better right heart function, was associated with a lower onset of MACE. In particular, a 1 mm increase in the TAPSE value is associated with a 33% reduction in the risk of developing a MACE. The analysis of the processed ROC curve and the measurement of the relative AUC have also demonstrated the accuracy of TAPSE as a predictor of MACE onset. The latter, on the other hand, is not significantly affected by the functional parameters of the left heart. Rather than FAC, RV strain or other RV function parameters, we chose TAPSE because it is easily detectable and reproducible in real‐life routinary echocardiographic evaluation of COPD patients with eventual cardiovascular comorbidities. Therefore, these results can be possibly explained by considering that several risk factors for COPD and MACE are similar. In this regard, it is noteworthy that smoking, diabetes and other metabolic dysfunctions such as hypercholesterolemia are frequently associated with both COPD and MACE, rather than RV function.

Another data that emerged from our study concerns the intake of inhalation therapy with LABA/LAMA, which reduces the risk of developing MACE by 19%, so that patients under dual inhaled therapy have a better prognosis than patients treated with single inhaled therapy. This result is quite interesting. Indeed, it is likely that more symptomatic COPD patients often complain of decreased RV function. On the other hand, by significantly decreasing lung hyperinflation, LABA/LAMA dual bronchodilation probably provides relevant benefits also with regard to cardiac outcomes. Independent predictors of MACE onset were found to be the presence of cardio‐metabolic risk factors, such as diabetes mellitus and an increased UA value, as well as the presence of higher S‐PAP values. In particular, our present results strongly suggest that TAPSE and S‐PAP are potentially crucial for prediction of MACE onset. In our opinion, this association represents the major strength of this study.

The presence of diabetes increased by more than double the risk of MACE in COPD patients. Emerging data also show that diabetes mellitus is associated with right heart dysfunction; the results of a previous study conducted by our group have indeed shown that in patients with newly diagnosed and never treated hypertension, subjected to an oral glucose load curve and with glycaemic values at one hour ≥155 mg/dl, with intolerance to carbon hydrates or diabetics, worse right heart function parameters were found (i.e., lower TAPSE and RVFAC values compared with the group of patients with normal glucose tolerance and glucose at one hour <155 mg/dl), as well as higher values of S‐PAP and PVR.^29^ These data therefore indicate that even before the onset of diabetes mellitus, glucose metabolism disorders can have a significant impact not only on the left heart sections but also on the right heart. Hyperglycaemia and insulin resistance also promote a pro‐inflammatory state with increased oxidative stress, mitochondrial dysfunction and increased levels of advanced glycation end‐products (AGEs), reduction in insulin‐like growth factor‐1 (IGF‐1) and activation of RAAS, all mechanisms that ultimately lead to fibrosis and myocardial damage.^30^, ^31^ Chronically elevated glucose levels can also be the cause of microangiopathy of the alveolar capillaries and pulmonary arterioles, which could justify an increase in the afterload of the RV and therefore, in the long run, the dysfunction of the latter.^32^ This is of great relevance since right heart dysfunction can modify the course and prognosis of several clinical conditions and diseases such as COPD, as observed in the present study. Additionally, our univariate analysis suggests that high BMI values are associated with a worse prognosis of COPD. However, this result does not appear to contradict the so‐called ‘obesity paradox’. Indeed, true obesity can reasonably be a poor prognostic factor also for COPD. What can be really protective for COPD patients is not a non‐specific high BMI value, but rather the preservation of a sufficient low‐fat body mass, which mainly reflects the entity of the striated components of muscle tissue.

**FIGURE 5:**
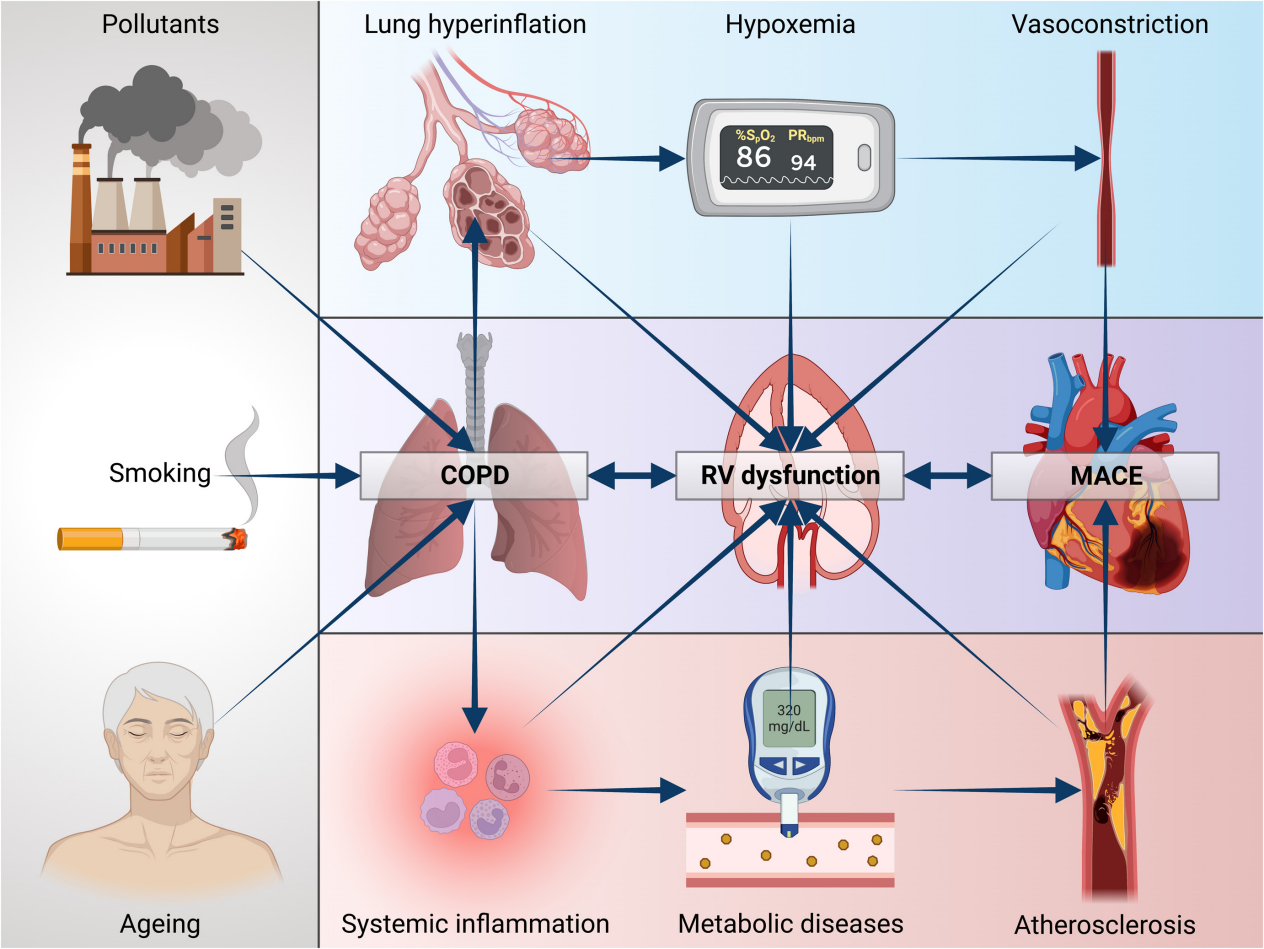
Possible pathophysiological mechanisms of right ventricular failure in COPD. COPD, chronic obstructive pulmonary disease; RV, right ventricle. Created with BioRender.com.

Furthermore, we herein demonstrate the influence of another parameter, namely UA, on the prognosis of our population with COPD; in fact, it was observed that in these patients a 1 mg/dl‐increase of UA resulted in a 12.5% increased risk of developing MACE. The role of UA in subjects with COPD in the initial phase of the disease was also analysed in a previous study carried out by our group, which documented that UA is an independent predictor of the onset of renal dysfunction as well as of the rapid decline in renal function in these patients; the 1 mg/dl increase in serum UA values increased the risk of the incidence of chronic kidney disease by 14.8%.^33^ Considering the high frequency of the disease, the practicality of UA as a biomarker, and the evidence of their correlation, several studies, also including that one performed by Bartziokas et al., have used UA as a marker to identify patients with high‐risk COPD.^34^ A study carried out by Zhang et al. compared two groups of COPD patients, one of whom also presented at the same time hyperuricaemia, and the latter, according to Kaplan–Meier curves, showed a higher risk of mortality.^35^ UA is considered one of the main antioxidant factors present in plasma; however, its potential pro‐oxidant activity can become prevalent when an increase in its serum values is associated with pathologic situations capable of inducing a condition of increased oxidative stress, including COPD as in our case.^36^ A significant increase in chronic oxidative stress was detected in COPD patients, with a concomitant decrease in the production of anti‐ageing molecules such as sirtuins.^37^ These mechanisms characterize various other chronic diseases, such as cardiovascular, metabolic and neurodegenerative disorders; these conditions are associated with an increased cellular senescence due to shared mechanisms, such as mitochondrial dysfunction, epigenetic modifications, alteration of normal micro‐RNA profiles, shortening of telomeres and activation of PI3K/AKT mTOR signalling. The aforementioned molecular processes configure the so‐called ‘inflammaging’ framework, which results in the activation of pro‐inflammatory transcription factors (NF‐κB), as well as secretion of cytokines (IL‐1 and IL‐6), chemokines (CXCL1/8, CCL2) and growth factors (TGF‐β).^38^ Therefore, these mechanisms generate a pro‐inflammatory and pro‐oxidant loop capable of self‐maintaining and progressively strengthening inflammation.^39^ Finally, the results of our study show that an increase in S‐PAP values of 5 mmHg increased the risk of MACE by 22%, which is consistent with most of the studies conducted in COPD patients, which have demonstrated that the severity of pulmonary hypertension and the development of cor pulmonale are among the main factors involved in the mortality of these patients. However, despite all the evidence showing the relationship between hypoxaemia, which mainly occurs in severe COPD, and pulmonary hypertension with consequent right heart dysfunction, recent studies have led to the detection of structural and functional impairments of the RV also in patients with mild COPD, not hypoxemic and in the absence of pulmonary hypertension. One of these investigations evaluated the structural and functional features of the RV in 98 patients with COPD, subdivided into two groups based on the presence or absence of pulmonary hypertension, diagnosed by catheterization of the right heart; these patients were subsequently compared with 34 healthy controls.^40^ In comparison with the control group, a significant reduction in systolic function as well as a condition of right ventricular hypertrophy were also observed in patients who did not have pulmonary hypertension.^40^ These findings suggest that cor pulmonale may actually represent a continuum of right heart disease, which can begin long before the onset of resting pulmonary hypertension (Figure 5). In this regard, the lack of two potentially relevant endpoints, including hospitalization numbers due to heart failure and high values of B‐type natriuretic peptide, should be considered as a major limitation of the present investigation.

In light of these results, it should be essential to implement and optimize a combined therapeutic strategy aimed to improve both cardiac and respiratory function, as well as to evaluate coexisting risk factors, in order to reduce comorbidity and mortality in COPD patients, thus improving the overall prognosis.

## AUTHOR CONTRIBUTIONS

All authors contributed to design and carry out the study protocol as well as to write the text.

## FUNDING INFORMATION

This research received no specific grant from any funding agency in the public, commercial or not‐for‐profit sectors.

## CONFLICT OF INTEREST

The authors declare that there is no conflict of interest.

## Data Availability

The raw data supporting the conclusions of this article will be made available by the authors, without undue reservation.
